# Is male infertility associated with increased oxidative stress in seminal plasma? A-meta analysis

**DOI:** 10.18632/oncotarget.25075

**Published:** 2018-05-11

**Authors:** Chao Huang, Xiyue Cao, Dejiang Pang, Chao Li, Qihui Luo, Yuanfeng Zou, Bin Feng, Lixia Li, Anchun Cheng, Zhengli Chen

**Affiliations:** ^1^ Laboratory of Experimental Animal Disease Model, College of Veterinary Medicine, Sichuan Agricultural University, Chengdu 611130, China; ^2^ Key Laboratory of Animal Disease and Human Health of Sichuan Province, College of Veterinary Medicine, Sichuan Agricultural University, Chengdu 611130, China; ^3^ Department of Biochemistry and Molecular Biology, West China School of Preclinical and Forensic Medicine, West China Hospital, Sichuan University, Chengdu 610041, China; ^4^ Animal Nutrition Institute, Sichuan Agricultural University, Chengdu 611130, China

**Keywords:** oxidative stress, antioxidants, male infertility, meta-analysis, markers

## Abstract

**Objectives:**

We conducted a systematic review and meta-analysis of observational case-control studies to evaluate markers of oxidative stress in seminal plasma of patients with male infertility.

**Background:**

Current evidence links oxidative stress to male infertility, in which multiple markers of oxidative stress have been widely detected, publishing inconsistent results with regard to the role of oxidative stress markers in the evaluation of male infertility. Therefore, a systematic review and meta-analysis on this issue is necessary.

**Results:**

From the 1024 articles initially retrieved, 65 studies were included in our meta-analysis with 11 oxidative stress markers, containing 3819 male infertility patients and 2012 controls. The concentrations of malondialdehyde (SMD = 1.86, *p* < 0.00001), NO (SMD = 0.89, *p* = 0.001), carbonyl protein (SMD = 2.09, *p* < 0.00001) in seminal plasma were significantly higher in infertility patients. The concentrations of GSH (SMD = –1.68, *p* < 0.00001), vitamin C (SMD = –1.12, *p* < 0.00001), and vitamin E (SMD = –1.48, *p* = 0.003), as well as the activities of catalase (SMD = –1.91, *p* < 0.0001), glutathione peroxidase (SMD = –1.96, *p* = 0.0002) and glutathione-S-transferase (SMD = –1.62, *p* = 0.009) declined remarkably, resulting in decreased total antioxidant capacity (SMD = –1.77, *p* < 0.00001). Besides, the activity of superoxide dismutase showed no statistical difference between infertility patients and controls (SMD = –0.51, *p* = 0.07).

**Conclusions:**

Our meta-analysis suggests that oxidative stress in seminal plasma resulting from decreased antioxidant defense are associated with male infertility.

**Methods:**

Using PubMed, EMBASE, CNKI, VIP, and Wanfang database, we searched for literature reporting the detection of oxidative stress markers in the seminal plasma of male infertility published up to June 2017. Standardized mean differences (SMDs) and 95% confidence intervals (95%CI) were calculated for the finally analysis.

## INTRODUCTION

Infertility has become an increasing medical problem affecting approximately 15% of couples worldwide, of which male factors account for almost 50% cases [1]. Male infertility is common due to deficiencies in the semen, so seminal analysis is a keystone for the assessment of male fertilization potential, according to which the semen deficiencies are often labeled as oligozoospermia, aspermia, hypospermia, azoospermia, teratospermia, asthenozoospermia, or even combinations of these [2]. Genetic problems, immunologic disorders, obstructive lesions, varicocele, cryptorchidism *etc.* are frequently involved in male infertility, but the exact mechanisms of many cases are not well known, making the therapy face lots of challenges [3–5]. As a result, growing interests are elicited in identifying more causes of male infertility.

In recent decades, extensive researches have reported an important role of oxidative stress in causing male infertility, suggesting that the pathology of infertility in 30–80% of infertile men is oxidative damage to spermatozoa [6–10]. Oxidative stress happens as a result of the excessively reactive oxygen species (ROS) or reactive nitrogen species (RNS) accumulation, due to either increased ROS/RNS generation or impaired ROS/RNS clearance. It will cause extensive sperm DNA damage [11], reduced sperm motility [12], decline in sperm fertilising ability [13], and defective sperm membrane integrity via lipid peroxidation [14], all of which are important mechanisms behind sperm dysfunction. The major sources of ROS/RNS in semen include activated leukocytes in the seminal plasma and the mitochondria in the spermatozoa, while leukocytes in seminal plasma produce 1,000 times more ROS/RNS than the spermatozoa does [15].

To counteract the effects of ROS/RNS, semen is proved to possess a large amount of antioxidants, thereby protecting gonadal cells and mature spermatozoa from oxidative damage, especially after leaving the testicles. The antioxidants in semen are present both in spermatozoa and seminal plasma, but most abundant in seminal plasma, because the amount of spermatozoa cytoplasm is low, making the antioxidant defense activity limited [16]. Seminal plasma is enriched with both enzymatic antioxidants, such as superoxide dismutase (SOD), catalase, glutathione peroxidase (GPX), glutathione S-transferase (GST), and non-enzymatic antioxidants such as glutathione, vitamin A, vitamin C, vitamin E, coenzyme Q10 etc. [17]. Deficiencies of enzymatic or non-enzymatic antioxidant systems in seminal plasma are widely associated with male infertility as the absence of any of these systems leads to the accumulation of excessive levels of ROS, resulting in impairment of both the structural and functional integrity of spermatozoa [18, 19]. However, previous studies addressing the oxidative stress status or detecting the markers of oxidative stress in seminal plasma of male infertile patients yielded controversial results, possibly due to the relatively small sample sizes of most of these studies, the different regions where the subjects were from, and the variability in the markers and assays used in them. In order to overcome these limitations, we conducted a systematic review and meta-analysis, and sought to rigorously evaluate current evidence from a large number of studies addressing the differences in markers of oxidative stress (MDA, TAC, SOD, Catalase, GPX, GST, Ve, Vc, NO, GSH and protein carbonyl) in seminal plasma between male infertile patients and controls.

## METHODS

This systematic review and meta-analysis was performed according to the preferred reporting items for systematic reviews and meta-analyses.

### Search strategies

A systematic review of the literature was conducted using PubMed, EMBASE, Chinese National Knowledge Infrastructure (CNKI), VIP information database, and Wanfang database. Studies reporting biomarkers of oxidative stress in the seminal plasma of infertile patients and controls published up to June 2017 were identified and analyzed. In addition, a hand search of the references of the retrieved articles and relevant reviews was performed to identify other potentially eligible studies. The following search strategy was used for PubMed, and was modified to suit other databases to include potentially relevant reports. Disagreements were resolved by discussion or consensus of a fourth reviewer (Z.C.).

1. infertility/ or infertile/ or subfertile/ or subfertility/ or sterility

2. oxidative stress/ or antioxidant/ or reactive oxygen species/ or reactive nitrogen species/ or free radicals/ or nitric oxide

3. total antioxidant capacity/ or total antioxidant power/ or total oxidant status/ or total antioxidant status

4. oxidative damages/ or protein carbonyl/ or protein carbonylation/ or lipid peroxidation/ or malondialdehyde/ or 8-hydroxydeoxyguanosine/ or thiobarbituric acid reactive substances/ or asymmetric dimethylarginine

5. xanthine oxidase/ or superoxide dismutase/ or glutathione/ or glutathione peroxidase/ or glutathione transferase/ or catalase

6. homocysteine/ or vitamin A/ or vitamin C/ or vitamin E/ or coenzyme Q10

7. 1 and 2 or 3–6

### Inclusion and exclusion criteria

Inclusion criteria included: 1) studies where oxidative stress biomarkers were detected in the seminal plasma of infertile patients and fertile controls; 2) the mean and standard deviation of the levels of oxidative stress markers could be obtained or calculated using the data provided therein;

Exclusion criteria included: 1) case reports, abstracts, reviews, or irrelevant to the current analysis; 2) animal studies and female infertility; 3) studies of infertile patients with testicular varicocele, leukocytospermia, infection, or on antioxidant supplement; 4) studies only reporting oxidative stress markers in non-seminal plasma; 5) studies with no control groups; 6) studies with data only in graphic form; 7) duplicate publications or studies with inappropriate outcome measure.

### Data extract

Two reviewers (C. H. and X.C.) independently performed data extraction using a standard data extraction form to determine eligibility for inclusion and data extract. Data was extracted for meta-analysis only when the markers were studied in three or more reports. The study with the largest sample size was included in the meta-analysis to avoid over-representation of cases when the same author had published two or more articles using the same or similar data within five years; otherwise, the latest study was included. The extracted data elements of this review included: 1) publication details: first author's last name, publication year, and country of the studied population; (2) study design; (3) characteristics of the studied population: sample size, age; (4) the mean and standard deviation of the oxidative stress markers. Disagreements were resolved by discussion or consensus with a third reviewer (Z.C.).

If the study provided medians and interquartile ranges instead of means and SDs, we imputed the means and SDs as described by Hozo *et al*. [20]. The combined mean and standard deviation for several subgroups in a study were computed as described by Borenstein *et al*. [21].

Missing or ambiguous data were solicited from authors. The corresponding author of each article received a copy of the completed validity and data-abstraction forms and was then requested to correct erroneous assessments and provide missing information when necessary.

### Quality assessment

The quality of the studies included in the meta-analysis were assessed according to a modification of the Newcastle-Ottawa Quality Assessment Scale (NOS) for case–control studies as described by Wells *et al*. [22]. The total possible score for each article is nine points; articles with more than five points were considered of high quality, and the others were considered of low quality.

To assess the quality of the pooled evidence, the Grading of Recommendations, Assessment, Development and Evaluation (GRADE) approach was adopted [23]. We developed Summary of Findings tables with the use of the GradePro software.

### Statistical analysis

Meta-analysis was performed using the Review Manager software (Vision 5.3, The Cochrane Collaboration, Oxford, United Kingdom). We used Hedge's g standardized mean differences (SMDs) as a measure of effect size because the outcomes of the studies were frequently measured by different assays and techniques. SMD becomes dimensionless and the scales become uniform across the different studies. Results were given as SMD and 95% confidence intervals (95%CI).

The statistical heterogeneity was determined by the value of the I^2^ statistic using Hedge's test. When the statistical heterogeneity was notable (I^2^ > 50%), an integrated effect was calculated with a random-effect model; otherwise, the fixed-effect model was used.

Publication bias was estimated by funnel plot asymmetry and Egger's regression test performed by Stata 14.0 software, when 10 or more studies were included in the same analysis [24]. A sensitivity analysis was also performed with Stata 14.0 software to evaluate the robustness in the conclusion of each analysis with over 10 or more studies [25]. All *P* values were evaluated using two-tailed tests, and *p* < 0.05 was set as statistically significant.

## RESULTS

**Figure 1:**
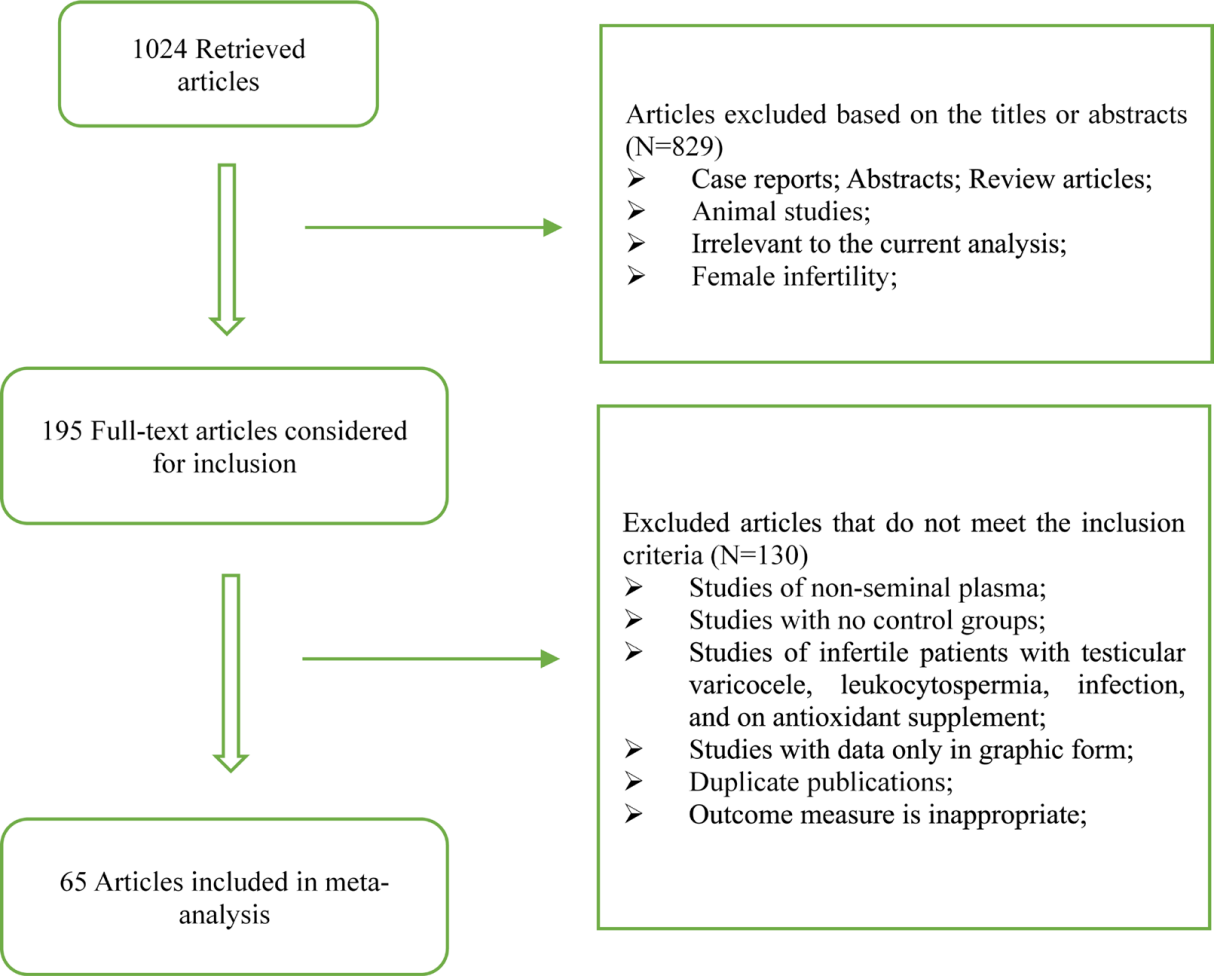
Flow diagram of the trial selection process

The flow chart of preferred reporting items for Systematic Review and Meta-Analysis was present in Figure 1. 1024 records were retrieved after the primary literature search. However, after screening the titles and abstracts, 829 studies were excluded because they were case reports, abstracts, review articles, animal studies, or irrelevant to the current analysis. The remaining 195 reports were detailed evaluated with the full text, and 130 studies were excluded according to the exclusion criteria and quality assessment. 65 articles were finally included in our analysis, which contained 33 from Asia and Oceania, 20 from Europe, 6 from Africa and 6 from North/South America. These included articles contained 11 oxidative stress markers, 2012 fertile controls and 3819 infertile patients.

The eligible studies for meta-analysis were shown in Table 1, and they were marked with NOS scores for the methodological quality assessment (Supplementary Figure 1). There were 51 studies of high quality (NOS > 5), and the remaining 14 were of low quality (NOS ≤ 5).

**Table 1:** Characteristics and quality assessment of the studies included in the meta-analysis

Author	Year	Country	Simple size (*n*)		Mean or range of age (years)		Oxidative stress biomarkers	NOS
			Fertility	Infertility	Fertility	Infertility		
Atig, *et al.* [26]	2017	Tunisia	50	100	34.81 ± 5.43	39.40 ± 5.20	MDA, SOD, GPX, CAT	8
Fafula, *et al.* [27]	2017	Ukraine	20	39	N/A	N/A	MDA, GPX, GST	7
Vatannejad, *et al.* [28]	2017	Iran	28	25	N/A	N/A	TAC	4
Fazeli, *et al.* [29]	2016	Iran	34	35	N/A	N/A	MDA, TAC	6
Micheli, *et al.* [30]	2016	Italy	14	16	22–44	22–40	MDA, GPX, CAT, GSH	8
Riaz, *et al.* [31]	2016	Pakistan	20	20	N/A	N/A	TAC	5
Roychoudhury, *et al.* [32]	2016	USA	46	279	37.57 ± 11.37	35.61 ± 6.06	TAC	6
Taken, *et al.* [33]	2016	Turkey	22	30	30.40 ± 4.97	29.90 ± 4.28	MDA, NO	8
El-Taieb, *et al.* [34]	2015	Egypt	20	30	33.5 ± 7.69	35.26 ± 10.07	MDA	8
Layali, *et al.* [35]	2015	Iran	12	25	31.21 ± 4.07	29 ± 3.37	MDA, TAC	8
Tamilselvan, *et al.* [36]	2015	India	10	10	30.20 ± 2.74	30.10 ± 2.56	SOD, GPX	6
Yousefniapasha, *et al.* [37]	2015	Iran	33	30	24–38	24–38	TAC, NO	8
zhao, *et al.* [38]	2015	China	40	40	24–36	25–39	SOD	6
Al-Azzawie, *et al.* [39]	2014	Iraq	30	50	N/A	23–45	MDA, SOD, GPX, CAT, GSH, VC, VE	7
Collodel, *et al.* [40]	2014	Italy	14	20	24–45	24–40	MDA	8
Eroglu, *et al.* [41]	2014	Turkey	15	44	32.5 ± 5.3	31.2 ± 4.9	TAC	8
Mostafa, *et al.* [13]	2014	Egypt	20	40	30.15 ± 6.8	30.9 ± 5.1	MDA, GPX	8
Türk, *et al.* [42]	2014	Estonia	27	32	31.0 ± 2.0	31.0 ± 1.5	TAC, GPX	8
Jiao, *et al.* [43]	2013	China	21	72	25–30	20–35	MDA, TAC	7
Kullisaar, *et al.* [44]	2013	Estonia	27	32	30.0 ± 5.2	32.2 ± 6.6	NO	8
Mehrotra, *et al.* [45]	2013	India	15	40	30.79 ± 1.83	36.53 ± 3.39	TAC, MDA	8
Benedetti, *et al.* [46]	2012	Italy	12	31	40 ± 5	41 ± 6	TAC, MDA, VE	8
Gao, *et al.* [47]	2012	China	100	120	29 ± 3.1	30 ± 3.3	MDA, SOD	7
Khalil, *et al.* [48]	2012	Egypt	40	80	20–35	20–35	SOD, GPX, GST	7
Shamsi, *et al.* [49]	2012	India	76	93	29.14 ± 1.78	31.75 ± 4.82	TAC	8
Shete, *et al.* [50]	2012	India	30	50	21–58	21–58	GSH	7
You, *et al.* [51]	2012	China	50	30	27.5 ± 4.7	27 ± 4.5	MDA	6
Badade, *et al.* [52]	2011	India	30	30	21–45	21–45	MDA, SOD	8
Li, *et al.* [53]	2011	China	30	30	24–36	24–38	MDA, SOD	5
Tang, *et al.* [54]	2011	China	30	65	29.2 ± 8.7	26.5 ± 5.6	MDA, NO	8
Abdul-rasbeed, *et al.* [55]	2010	Iraq	39	60	31.87 ± 3.76	31.94 ± 4.21	MDA	7
Ahmad, *et al.* [56]	2010	India	75	75	25–40	25–40	MDA, SOD, GPX, CP, GSH, VC, VE	8
Abdallah, *et al.* [57]	2009	Tunisia	9	93	N/A	N/A	MDA, SOD, CAT	5
Elshal, *et al.* [58]	2009	Egypt	16	70	<45	<45	MDA, SOD, CAT, GSH	7
El-Taieb, *et al.* [59]	2009	Austria	10	20	35.6 ± 5.72	38.85 ± 10.52	CP	6
Kumar, *et al.* [60]	2009	India	30	33	31	30.5	MDA, SOD, GPX, CAT	8
Mahfouz, *et al.* [61]	2009	USA	55	42	N/A	N/A	TAC	5
Nouri, *et al.* [62]	2008	Iran	40	60	37.1 ± 4	34.2 ± 3.4	MDA, VC, VE	7
Tavilani, *et al.* [63]	2008	Iran	15	30	20–40	20–40	SOD, CAT, GPX	8
Amiri, *et al.* [64]	2007	Iran	70	45	31.40 ± 4.60	33.20 ± 5.50	NO	6
Aydemir, *et al.* [65]	2007	Turkey	60	52	40.00 ± 7.00	39.12 ± 5.94	MDA, GSH, CP	8
Khosrowbeygi, *et al.* [8]	2007	Iran	16	46	32.06 ± 3.91	33.81 ± 5.84	TAC, SOD, CAT	8
Kiziler, *et al.* [66]	2007	Turkey	45	50	40.22 ± 9.0	39.54 ± 6.5	MDA, CP, GSH	8
Murawski, *et al.* [67]	2007	Poland	5	10	N/A	N/A	SOD	4
Verit, *et al.* [68]	2006	Turkey	20	30	31.60 ± 5.43	31.66 ± 5.06	TAC	5
Mehraban, *et al.* [69]	2005	Iran	40	40	27.9 ± 4.44	30.4 ± 5.17	NO	8
Shi, *et al.* [70]	2005	China	28	113	26–38	25–40	TAC	5
Tavilani, *et al.* [71]	2005	Iran	15	35	N/A	N/A	MDA	6
Li, *et al.* [72]	2004	China	18	81	29.4 ± 3.7	30.4 ± 3.1	MDA	7
Koca, *et al.* [73]	2003	Turkey	20	30	N/A	N/A	TAC	7
Raijmakers, *et al.* [74]	2003	Netherlands	25	25	34.25 ± 3.75	33.50 ± 3.50	GSH	7
Giannattasio, *et al.* [75]	2002	Italy	14	20	25–37	28–37	GPX	7
Siciliano, *et al.* [76]	2001	Italy	25	90	20–40	20–40	TAC, SOD, CAT	6
Pasqualotto, *et al.* [77]	2000	USA	19	36	N/A	N/A	TAC	6
zini, *et al.* [6]	2000	Canada	12	105	43 ± 2	36 ± 1	SOD, CAT	8
Sharma, *et al.* [78]	1999	USA	24	28	N/A	N/A	TAC	7
Zhang, *et al.* [79]	1999	China	108	350	27.56 ± 2.19	28.15 ± 3.08	SOD	5
Alkan, *et al.* [80]	1997	Turkey	10	18	28.4 ± 0.7	32.6 ± 1.5	SOD, CAT, GPX	8
Lewis, *et al.* [81]	1997	UK	18	41	N/A	N/A	VC, VE	5
Kurpisz, *et al.* [82]	1996	Poland	10	63	N/A	N/A	SOD	6
Smith, *et al.* [83]	1996	Chile	15	101	N/A	25–45	TAC	4
Fan, *et al.* [84]	1995	China	15	55	24–34	26–35	SOD	5
Chen, *et al.* [85]	1994	China	137	245	29.8	32.3	SOD	4
Jeulin, *et al.* [86]	1989	France	11	14	N/A	N/A	CAT	5
Li, *et al.* [87]	1989	China	27	75	26–40	22–40	SOD	5

NOS: Newcastle–Ottawa Quality Assessment Scale; MDA: malondialdehyde; TAC: total antioxidant capacity; SOD: superoxide dismutase; CAT: catalase; GPX: glutathione peroxidase; GST: glutathione S-transferase; GSH: glutathione; NO: nitric oxide; CP: carbonyl protein; VC: vitamin C; VE: vitamin E; N/A: not available.

### Meta-analysis of malondialdehyde concentration

**Figure 2:**
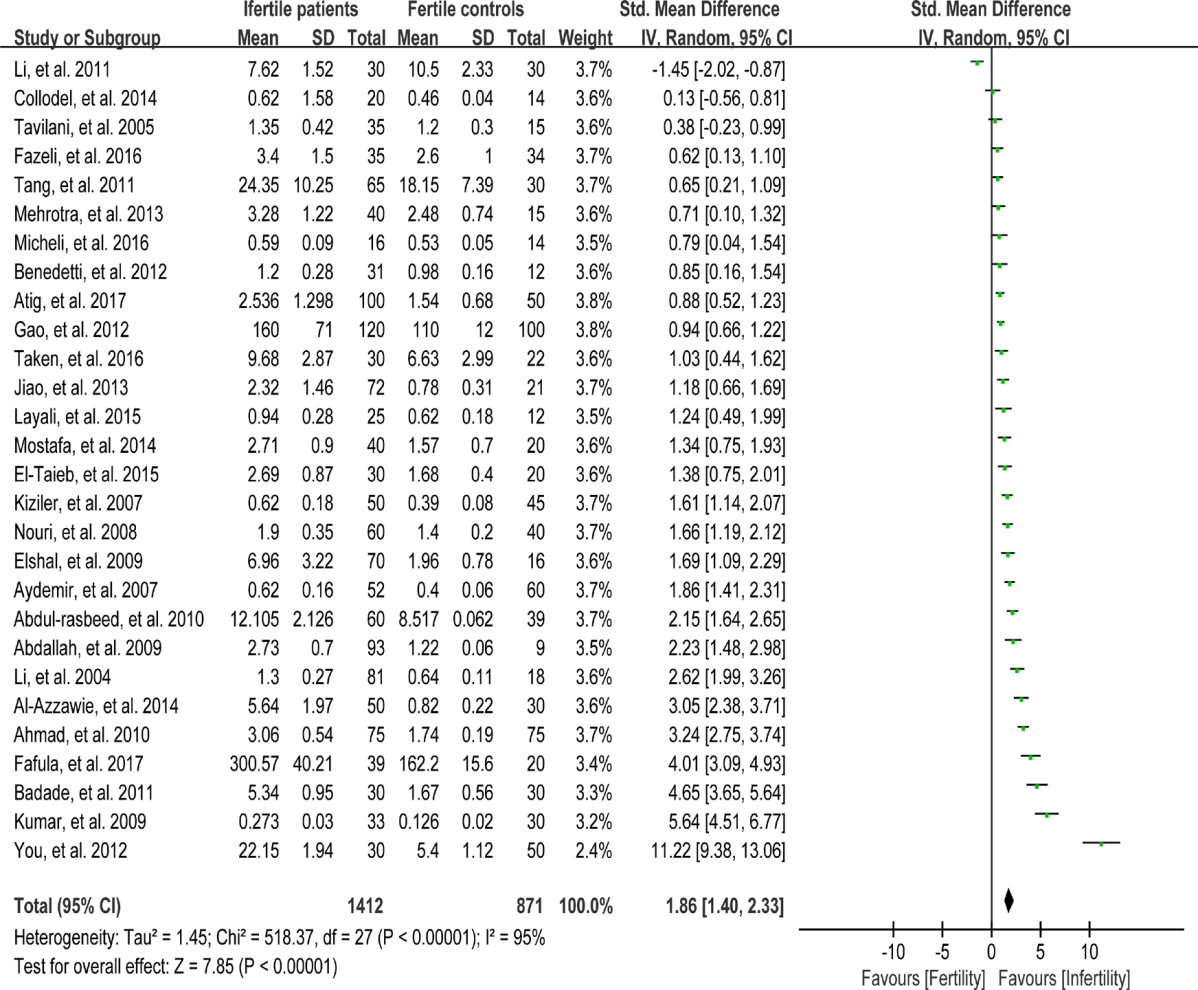
Meta-analysis of studies addressing MDA levels in seminal plasma of infertile patients and control subjects Results are shown as standardized mean differences (SMDs).

**Figure 3:**
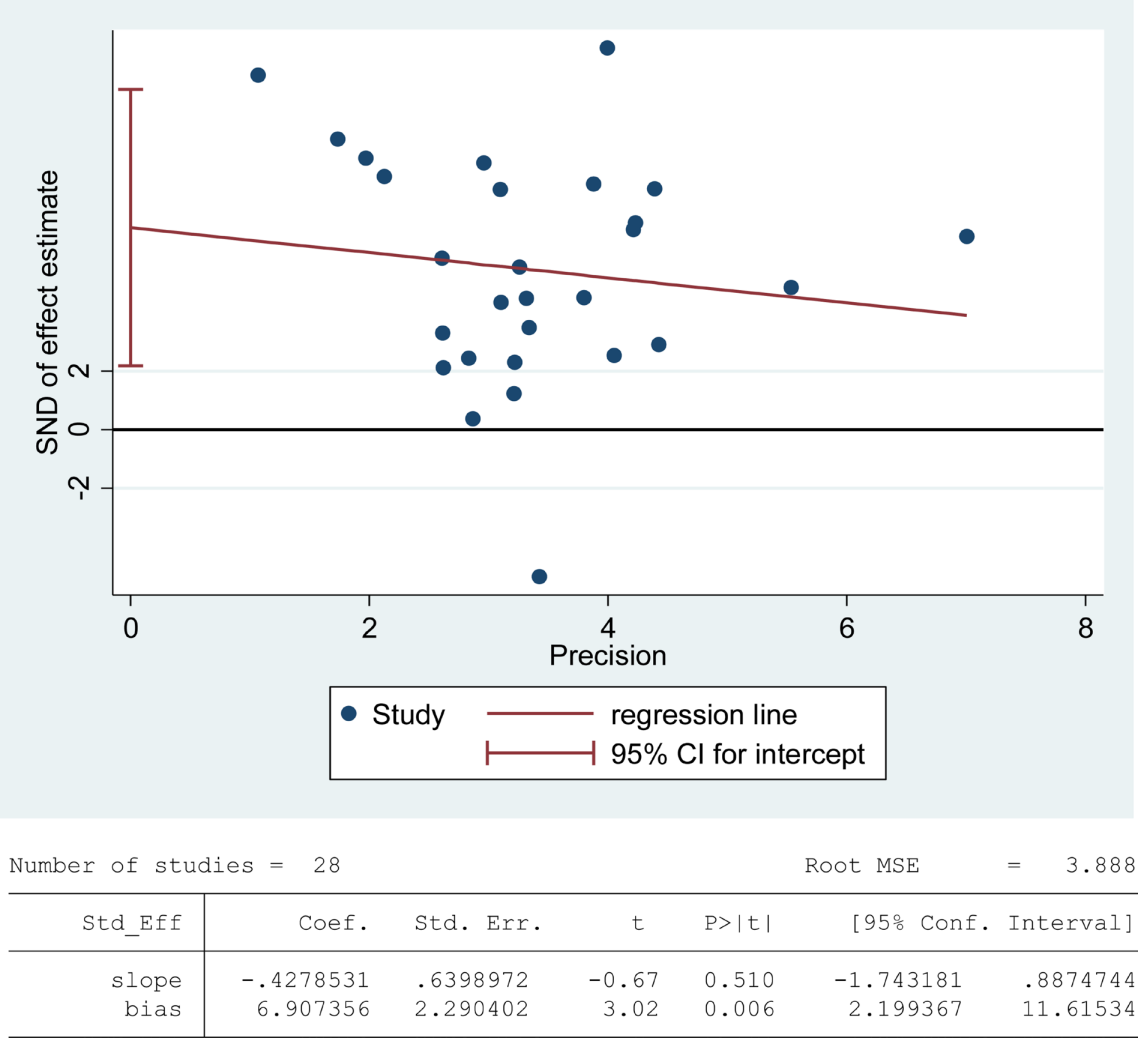
Evidence of publication bias in MDA concentration was assessed using funnel plot asymmetry and Egger's regression

Lipids are considered to be the most susceptible macromolecules to ROS and are present in sperm plasma membrane. Oxidative damage to lipids results in lipid peroxidation, and malondialdehyde (MDA) is one of the by-products of lipid peroxidation, which has been used in various biochemical assays to monitor the degree of peroxidative damage sustained by spermatozoa [88]. There were 28 articles including 1412 infertile patients and 871 fertile controls that were used to perform the meta-analysis of seminal plasma MDA concentration [13, 26, 27, 29, 30, 33–35, 39, 40, 43, 45–47, 51–58, 60, 62, 65, 66, 71, 72]. The statistical heterogeneity was notable (*I*^2^ = 95%), so the random-effect model was chosen to calculate the integrated effect, showing that the seminal plasma MDA concentrations in infertile patients were higher than those in control groups (SMD = 1.86, 95% CI: 1.40, 2.33, Z = 7.85, *p* < 0.00001; Figure 2). Egger's regression analysis displayed an evidence of publication bias for this marker, with no small study effect (Egger's regression intercept 6.907356, *t* = 2.29042, 95%CI 2.199367~11.61534, *P* = 0.006 < 0.05; Figure 3). We excluded the studies of low quality (NOS ≤ 5) [53, 57], yielding similar results and publication bias (SMD = 1.96, 95% CI: 1.51, 2.40, Z = 7.85, *p <* 0.00001; Egger's regression intercept 7.293577, *t* = 3.56, 95%CI 3.064078~11.52308, *P* = 0.002 < 0.05), so this conclusion needs to be interpreted cautiously. Meanwhile, a sensitivity analysis with a random effect model showed that the pooled estimate of the effect did not vary substantially with the exclusion of any of the studies, indicating the robustness of this result (Supplementary Figure 2).

### Meta-analysis of total antioxidant capacity

**Figure 4:**
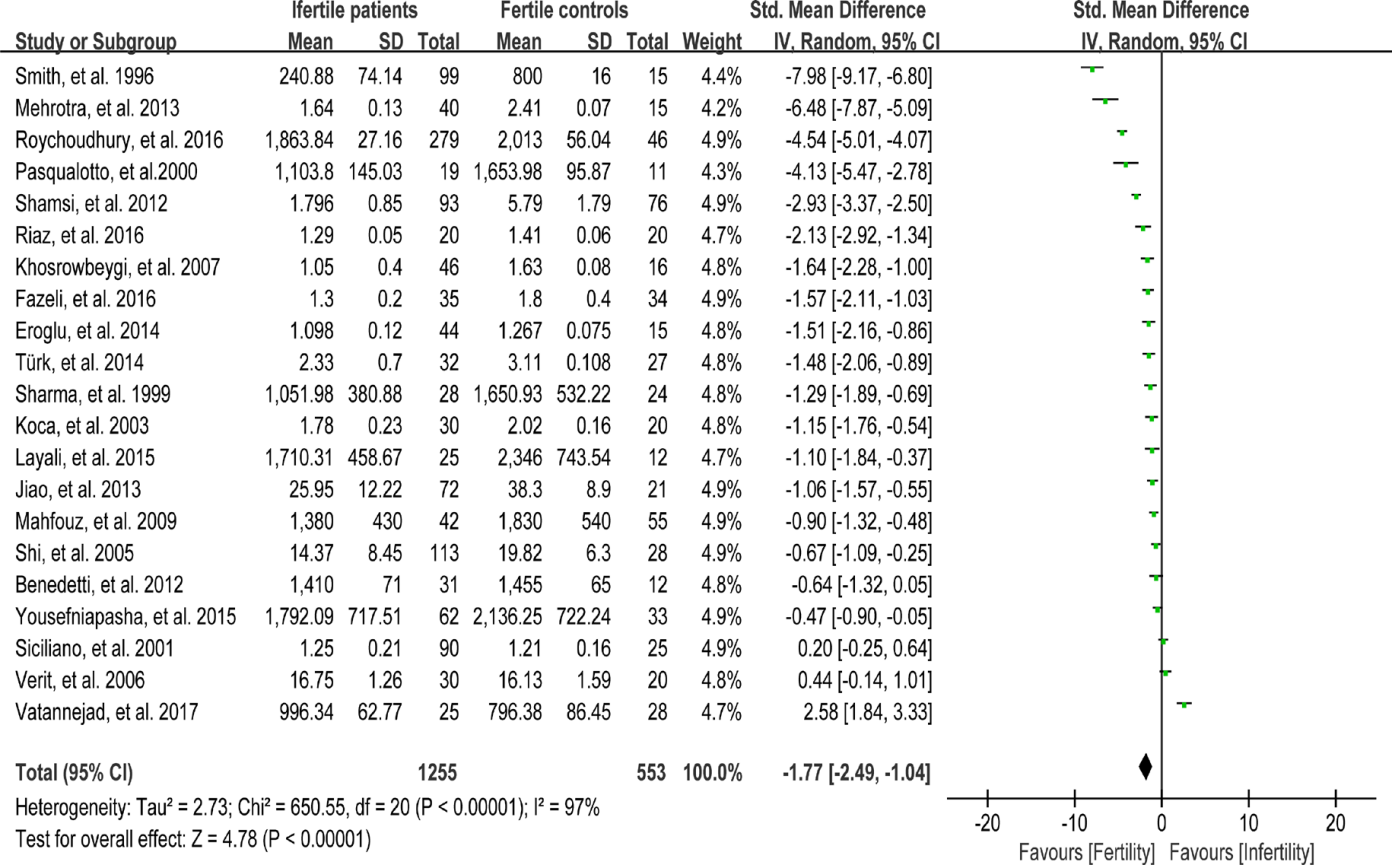
Meta-analysis of studies addressing TAC in seminal plasma of infertile patients and control subjects Results are shown as standardized mean differences (SMDs).

**Figure 5:**
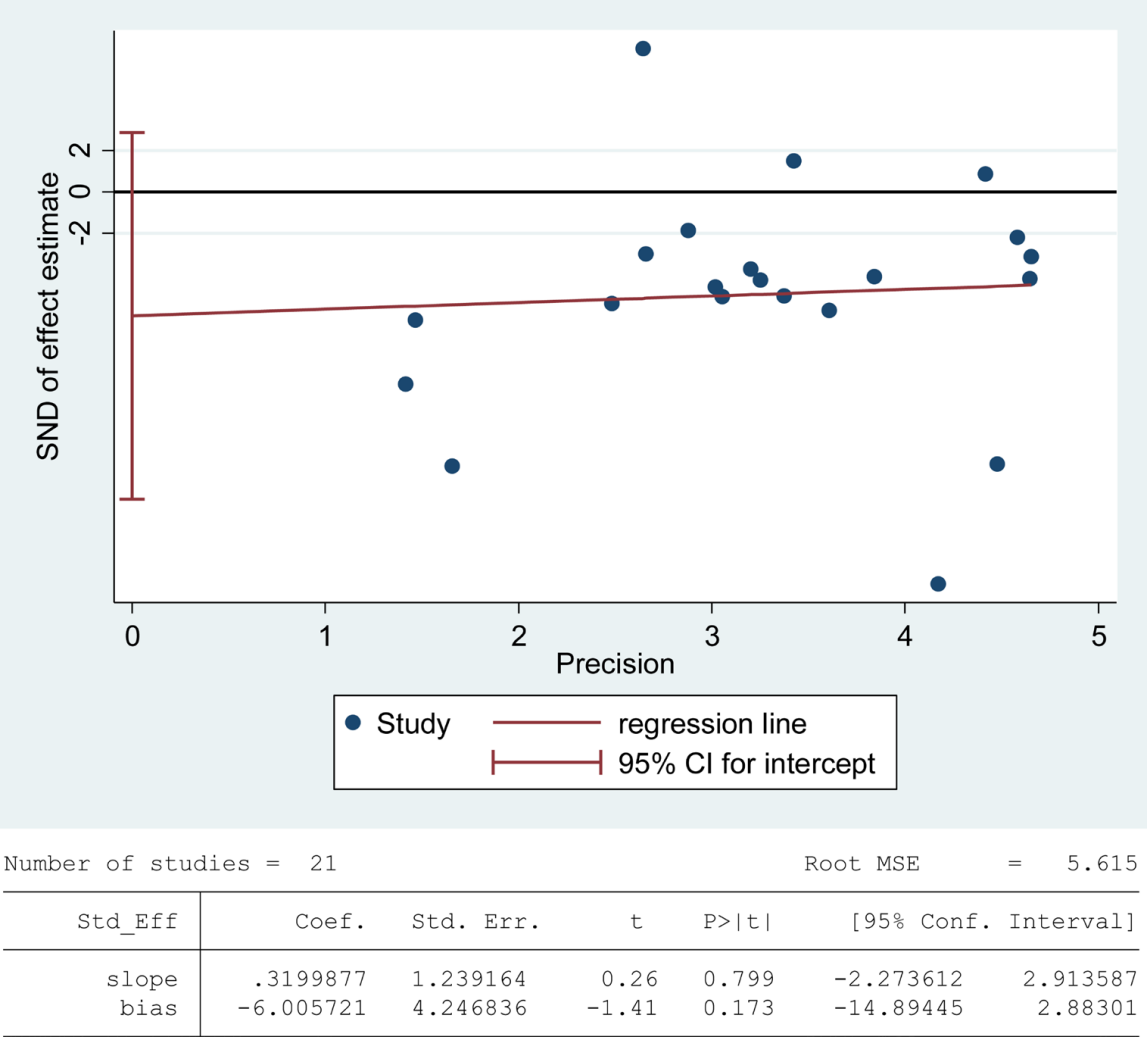
Evidence of publication bias in TAC was assessed using funnel plot asymmetry and Egger's regression

The total antioxidant capacity (TAC) of seminal plasma is measured by the levels of enzymatic and non-enzymatic antioxidants. Low TAC in semen is thought to be widely involved in male infertility [89]. Seminal plasma TAC was analyzed in meta-analysis with 21 articles containing 1255 infertile patients and 553 fertile controls [8, 28, 29, 31, 32, 35, 37, 41–43, 45, 46, 49, 61, 68, 70, 73, 76–78, 83]. The random-effect model was performed to calculate the integrated effect, because of the notable statistical heterogeneity (I^2^ = 97%). The TAC in seminal plasma of infertile patients was remarkably lower than that of fertile subjects (SMD = –1.77, 95% CI: –2.49, –1.04, Z = 4.78, *p* < 0.00001; Figure 4). Egger's test was performed to detect publication bias, and the results indicated no publication bias or small study effect, confirming the reliability of the conclusions (Egger's regression intercept –6.005721, *t* = –1.41, 95% CI –14.89445~2.88301, *P* = 0.173 > 0.05; Figure 5). A sensitivity analysis with a random effect model was performed to calculate the pooled estimate of the effect after exclusion of each of the studies, which showed no vary substantially results, indicating the robustness of this result (Supplementary Figure 3).

### Meta-analysis of superoxide dismutase activity

**Figure 6:**
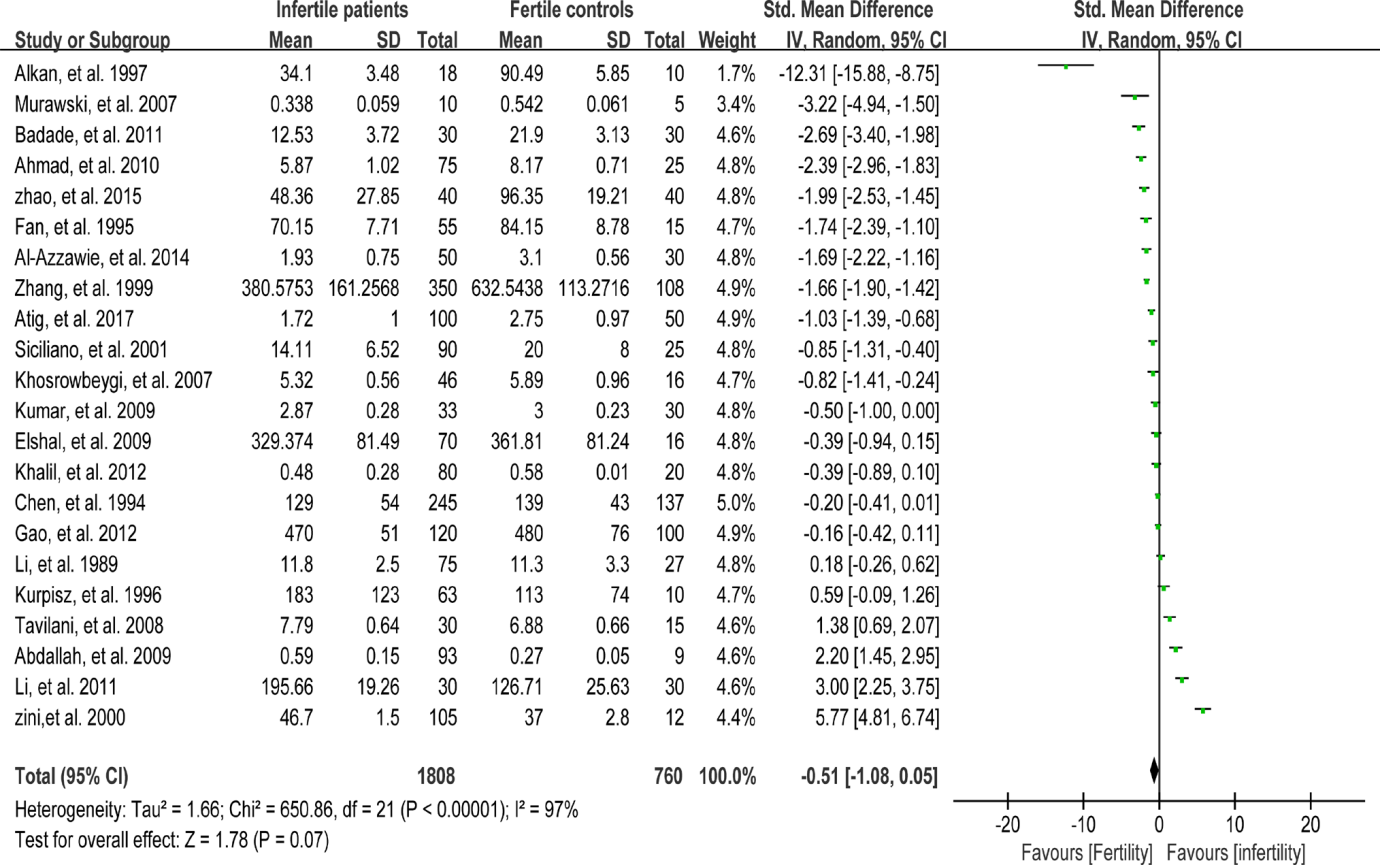
Meta-analysis of studies addressing SOD activity in seminal plasma of infertile patients and control subjects Results are shown as standardized mean differences (SMDs).

**Figure 7:**
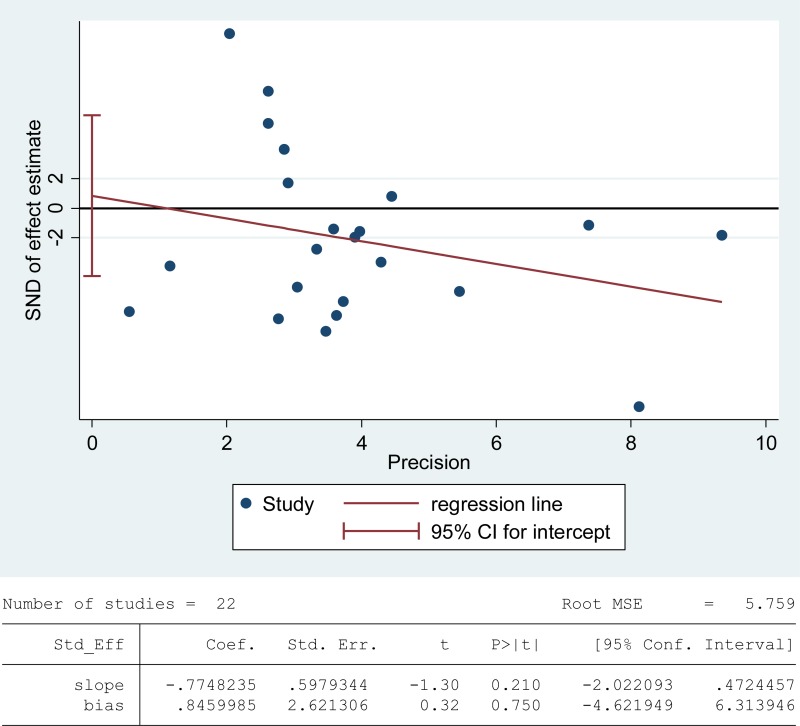
Evidence of publication bias in SOD activity was assessed using funnel plot asymmetry and Egger's regression

Superoxide dismutase (SOD) scavenges both intracellular and extracellular superoxide radicals and prevents the lipid peroxidation of plasma membrane [90]. Tween-one reports were included in the meta-analysis of SOD activity in seminal plasma with 1808 infertile patients and 760 fertile controls [6, 8, 26, 38, 39, 47, 48, 52, 53, 56–58, 60, 63, 67, 76, 79, 80, 82, 84, 85, 87]. There exhibited a notable statistical heterogeneity (I^2^ = 97%), and thus a random-effect model was performed to calculate the integrated effect. The results indicated no statistically significant difference of the superoxide dismutase (SOD) activity in infertile patients and fertile subjects (SMD = –0.51, 95% CI: –1.08, 0.05, Z = 1.78, *P* = 0.17 > 0.05; Figure 6). Meanwhile, the publication bias was evaluated through the Egger's test, showing no statistically significant evidence of publication bias or small study effect among these studies (Egger's regression intercept 0.8459985, *t* = 0.32, 95% CI –4.621949~6.313946, *P* = 0.750 > 0.05; Figure 7). The pooled estimate of the effect did not vary substantially displayed by a sensitivity analysis with a random effect model with the exclusion of each one of the studies, indicating the robustness of this result (Supplementary Figure 4).

### Meta-analysis of catalase activity

**Figure 8:**
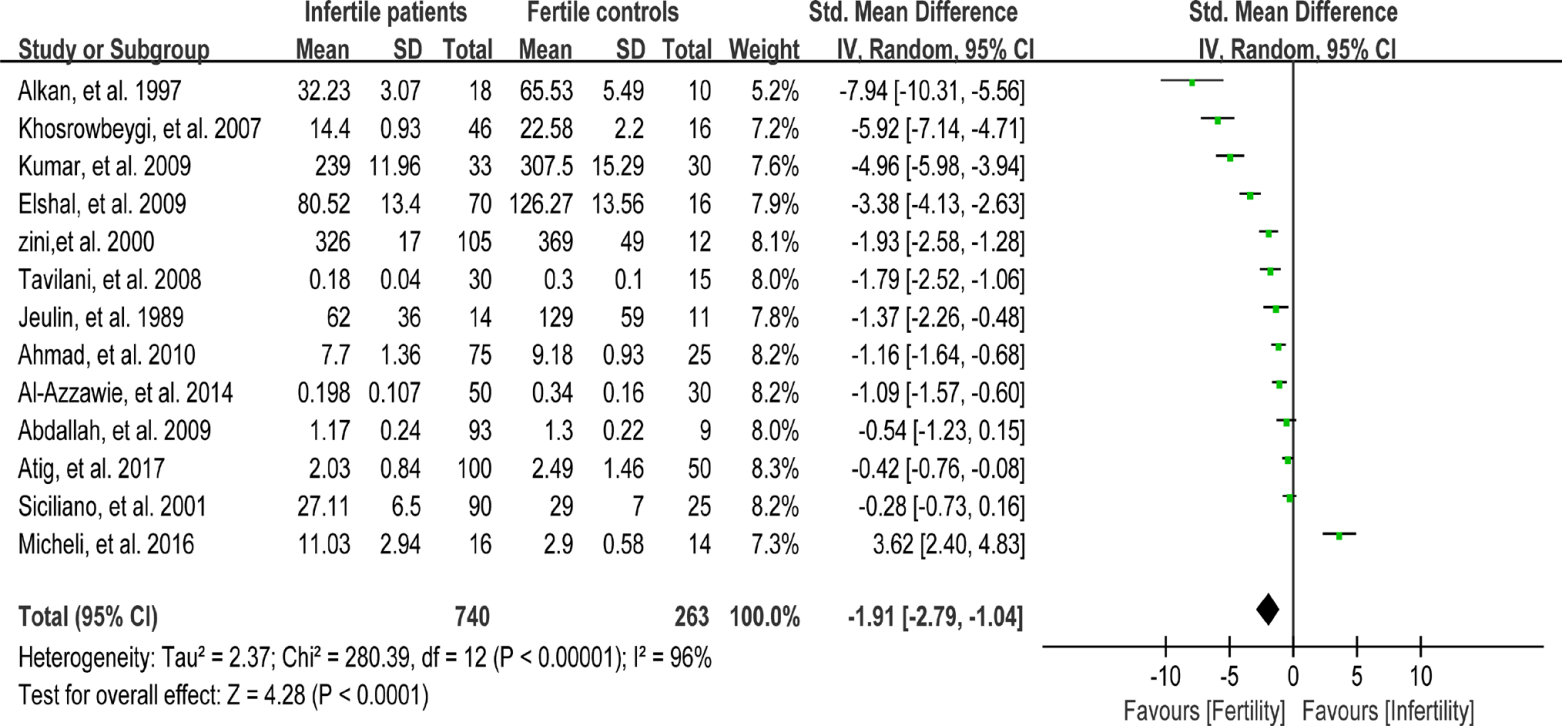
Meta-analysis of studies addressing catalase activity in seminal plasma of infertile patients and control subjects Results are shown as standardized mean differences (SMDs).

**Figure 9:**
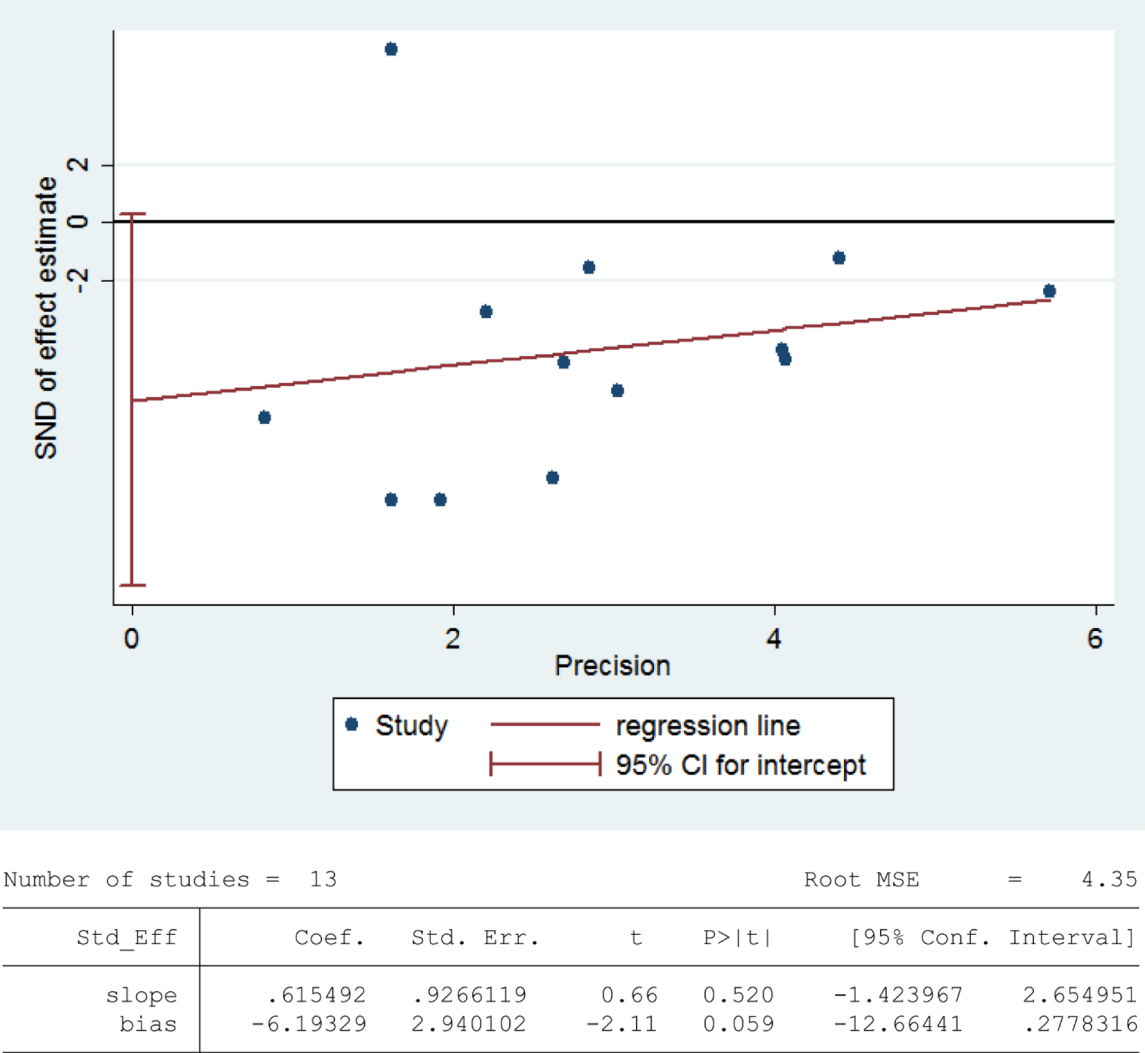
Evidence of publication bias in catalase activity was assessed using funnel plot asymmetry and Egger's regression

Catalase detoxifies both intracellular and extracellular H_2_O_2_ to water and oxygen [91]. In addition, catalase activates NO-induced sperm capacitation, which is a complex mechanism involving H_2_O_2_ [92]. Thirteen studies were included in the meta-analysis with 740 infertile patients and 263 fertile controls to analyze the catalase activity in seminal plasma [6, 8, 26, 30, 39, 56–58, 60, 63, 76, 80, 86]. The statistical heterogeneity was notable (I^2^ = 96%), and the integrated effect was calculated from a random-effect model hereby. Statistically lower catalase activity was present in infertile subjects than that in the control ones (SMD = –1.91, 95% CI: –2.79, –1.04, Z = 4.28, *p <* 0.0001; Figure 8). Egger's test was also performed to evaluate the publication bias, displaying no statistically significant evidence of publication bias and small study effect among these studies (Egger's regression intercept –6.19329, *t* = –2.11, 95%CI –12.66441~0.2778316, *P* = 0.059 > 0.05; Figure 9). A sensitivity analysis with a random effect model was performed to calculate the pooled estimate of the effect after exclusion of each of the studies, which showed no vary substantially results, indicating the robustness of this result (Supplementary Figure 5).

### Meta-analysis of glutathione peroxidase activity

**Figure 10:**
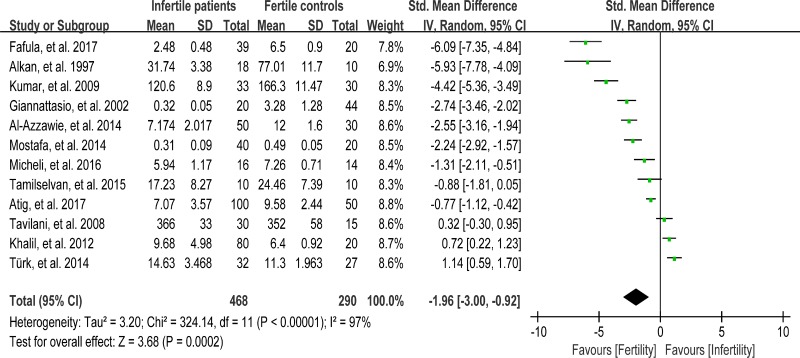
Meta-analysis of studies addressing GPX activity in seminal plasma of infertile patients and control subjects Results are shown as standardized mean differences (SMDs).

**Figure 11:**
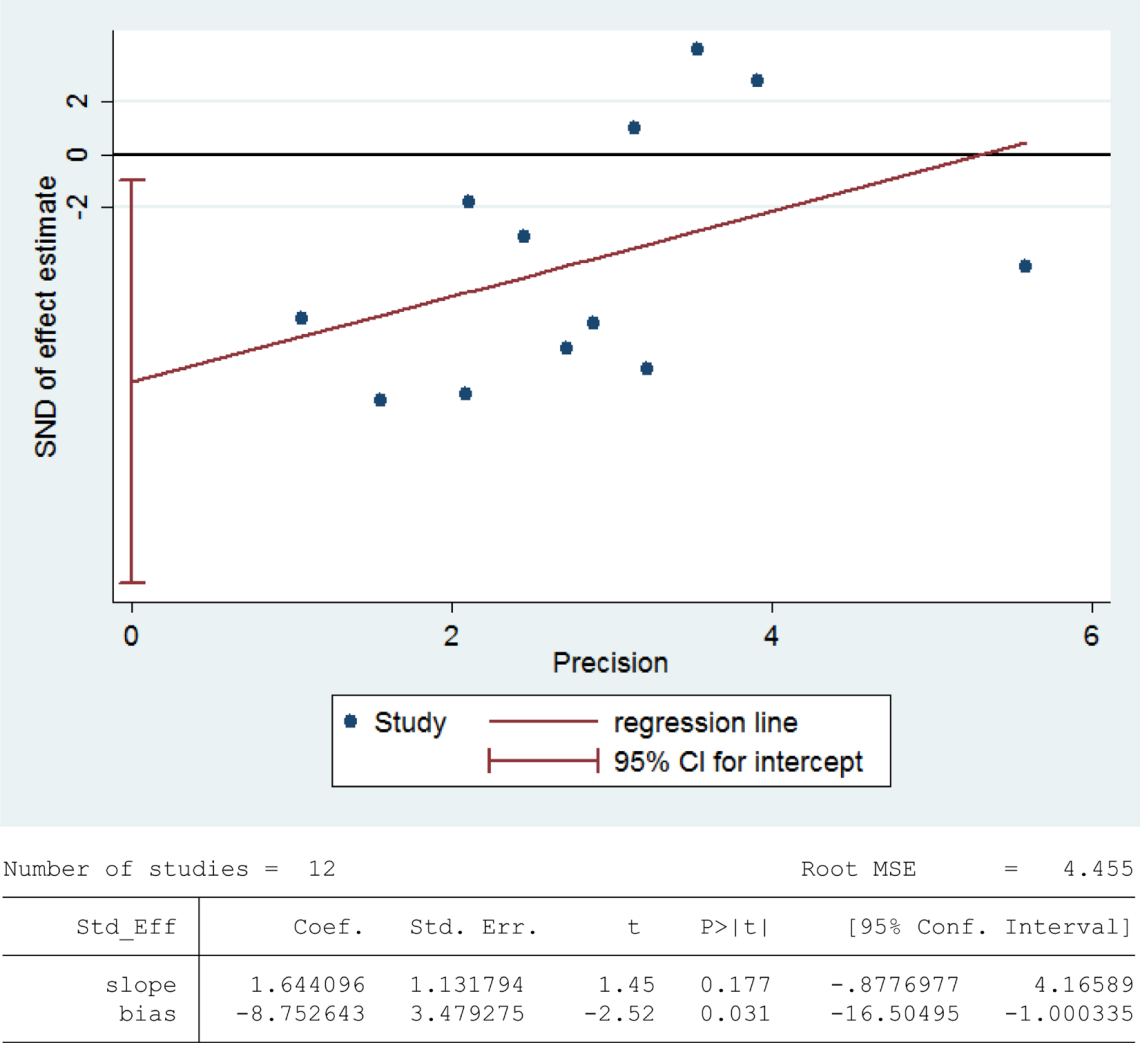
Evidence of publication bias in GPX activity was assessed using funnel plot asymmetry and Egger's regression

Glutathione peroxidase (GPX) reduces lipid hydroperoxides to their corresponding alcohols and free hydrogen peroxide to water [93]. 468 infertile patients and 290 fertile subjects from 12 studies were included in the meta-analysis to evaluate the GPX activity in seminal plasma [13, 26, 27, 30, 36, 39, 42, 48, 60, 63, 75, 80]. Notable statistical heterogeneity was present (I^2^ = 97%), so the integrated effect was calculated from a random-effect model. The GPX activity was significantly lower in infertile subjects when compared to fertile controls (SMD = –1.96, 95% CI: –3.00, –0.92, Z = 3.68, *P* = 0.0002 < 0.05; Figure 10). The publication bias was of significant existence according to an Egger's test (Egger's regression intercept –8.752643, *t* = –2.52, 95% CI –16.50495~-1.000335, *P* = 0.031 < 0.05; Figure 11), so this conclusion need to be interpreted cautiously. Meanwhile, the pooled estimate of the effect did not vary substantially displayed by a sensitivity analysis with a random effect model with the exclusion of each one of the studies, indicating the robustness of this result (Supplementary Figure 6).

### Meta-analysis of reduced glutathione concentration

**Figure 12:**
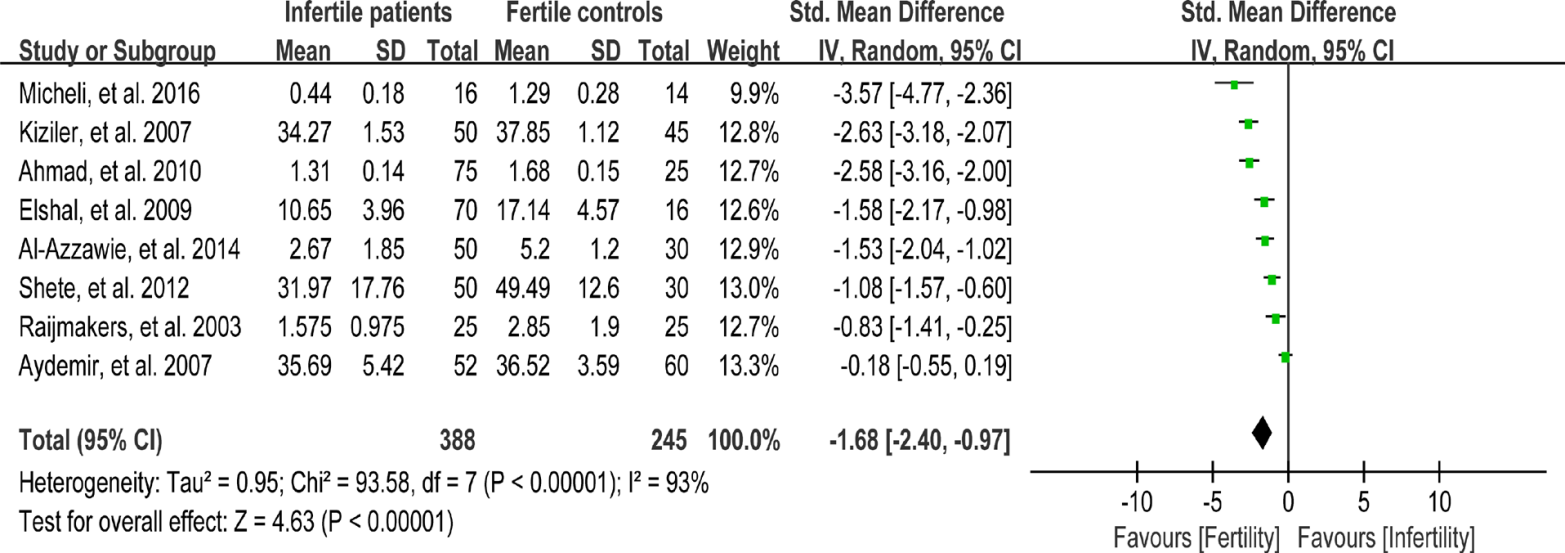
Meta-analysis of studies addressing reduced GSH concentration in seminal plasma of infertile patients and control subjects Results are shown as g standardized mean differences (SMDs).

Reduced glutathione (GSH) is able to react directly with cytotoxic aldehydes produced during lipid peroxidation, and thus protects the free sulphydryl groups on the sperm plasma membrane. Moreover, GSH acts to preserve SH groups of protein in the reduced state by means of disulfide interchange [94]. Eight studies were included in the meta-analysis to evaluate the reduced GSH concentration in seminal plasma, containing 388 infertile patients and 245 fertile controls [30, 39, 50, 56, 58, 65, 66, 74]. Remarkably evidence of heterogeneity was observed (I^2^ = 93%), so the integrated effect was calculated from a random-effect model. The GSH concentration in infertile patients was lower than that in fertile controls (SMD = –1.68, 95% CI: –2.40, –0.97, Z = 4.63, *p* < 0.00001; Figure 12).

### Meta-analysis of nitric oxide concentration

**Figure 13:**
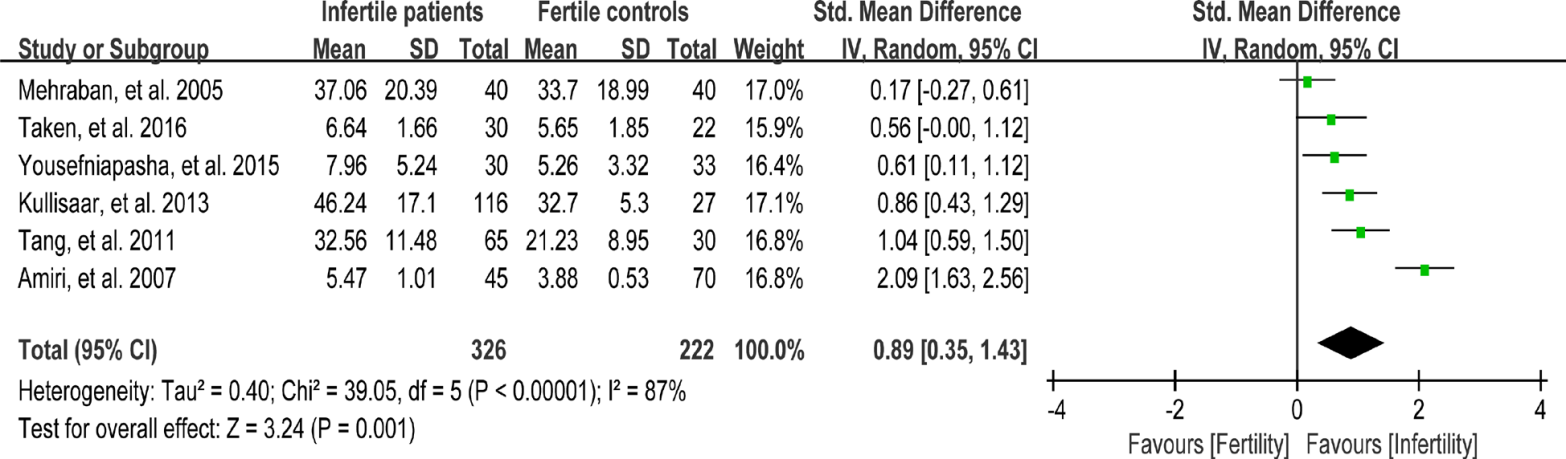
Meta-analysis of studies addressing NO concentration in seminal plasma of infertile patients and control subjects Results are shown as standardized mean differences (SMDs).

Nitric oxide (NO) is one of the reactive oxygen species that have been implicated in variety of physiologic cell signaling mechanisms in many tissues. Excessive NO could cause sperm hypomotility and reduce fertility in humans [95]. Six studies were included in the meta-analysis to evaluate the NO concentration in seminal plasma, containing 326 infertile patients and 222 fertile controls [33, 37, 44, 54, 64, 69]. The heterogeneity was notable (I^2^ = 87%), so the integrated effect was calculated from a random-effect model. The NO concentration was found to be much higher in infertile patients when compared with fertile controls (SMD = 0.89, 95% CI: 0.35, 1.43, Z = 3.24, *P* = 0.001 < 0.05; Figure 13). The controls from the two studies were of unproven fertility [54, 64], and exclusion of these studies yielded the similar results but with much less heterogeneity (NO concentration decreased in infertile patients; SMD = 0.55, 95% CI: 0.31, 0.79, Z = 4.52, *p <* 0.00001; *I* ^2^ = 40%; fixed-effect model), indicating that the fertility of controls may be the source of heterogeneity.

### Meta-analysis of vitamin E concentration

**Figure 14:**
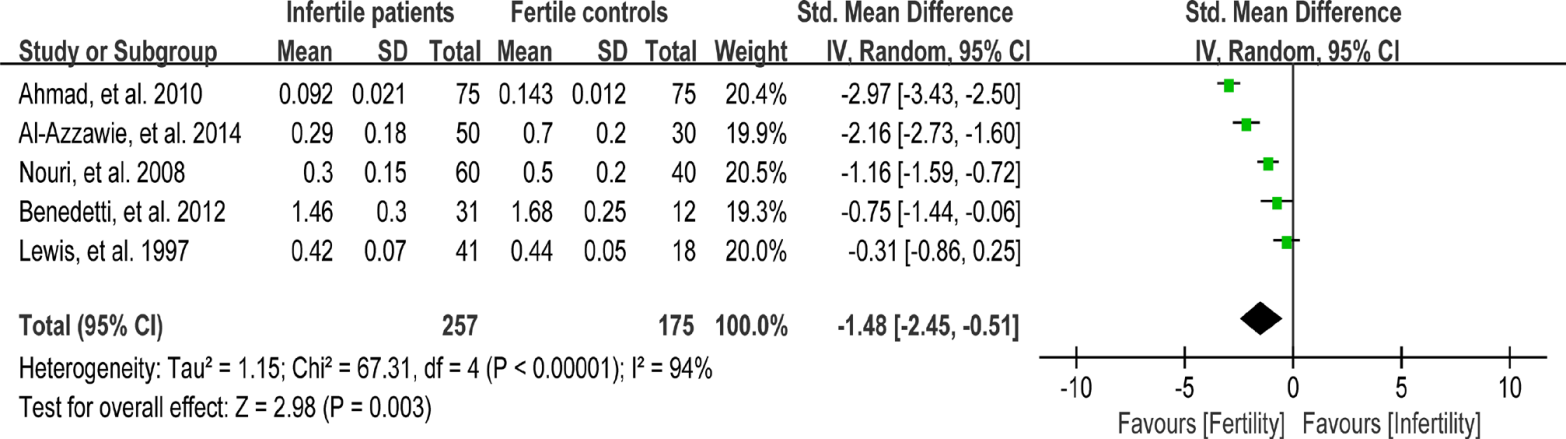
Meta-analysis of studies addressing VE concentration in seminal plasma of infertile patients and control subjects Results are shown as standardized mean differences (SMDs).

Vitamin E (VE) is a chain-breaking antioxidant in the sperm membranes and appears to scavenge superoxide, H_2_O_2_, and hydroxyl radicals [96]. Five studies were included in the meta-analysis to evaluate the VE concentration in seminal plasma, containing 257 infertile patients and 175 fertile controls [39, 46, 56, 62, 81]. The heterogeneity was notable (I^2^ = 94%), so a random-effect model was performed to calculate the integrated effect, showing that the VE concentration was statistically lower in infertile patients than that in fertile controls (SMD = –1.48, 95% CI: –2.45, –0.51, Z = 2.98, *P* = 0.003 < 0.05; Figure 14).

### Meta-analysis of vitamin C concentration

**Figure 15:**
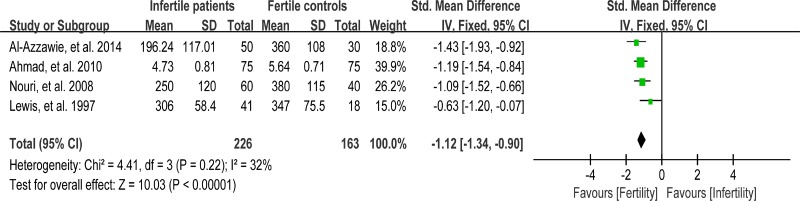
Meta-analysis of studies addressing VC concentration in seminal plasma of infertile patients and control subjects Results are shown as standardized mean differences (SMDs).

Vitamin C (VC) is also an important chain-breaking antioxidant that can neutralize hydroxyl, superoxide, and hydrogen peroxide radicals, and prevent sperm agglutination [96]. Four studies were included in the meta-analysis to evaluate the VC concentration in seminal plasma, containing 226 infertile patients and 163 fertile controls [39, 56, 62, 81]. A fixed-effect model was performed as there was no evidence of heterogeneity among these studies (*I* ^2^ = 32%), showing that the VC concentration was statistically lower in infertile patients than that in fertile controls (SMD = –1.12, 95% CI: –1.34, –0.90, Z = 10.03, *p* < 0.00001; Figure 15).

### Meta-analysis of carbonyl protein concentration

**Figure 16:**
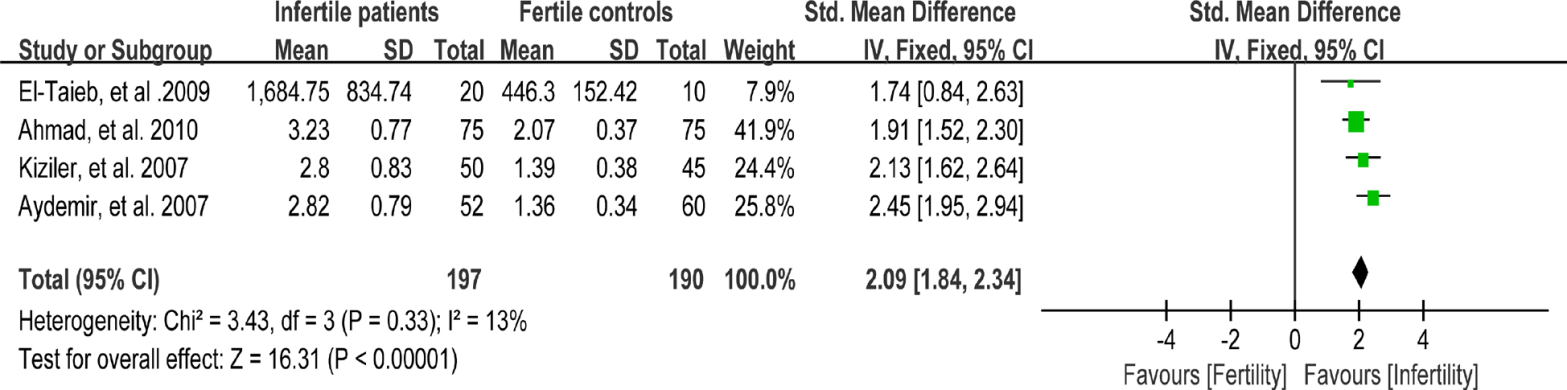
Meta-analysis of studies addressing carbonyl protein concentration in seminal plasma of infertile patients and control subjects Results are shown as standardized mean differences (SMDs).

Protein carbonyl content is actually the most universal indicator and by far the most commonly used marker of protein oxidation [97]. Accumulation of protein carbonyls has been observed in many human diseases including Alzheimer's disease (AD), diabetes, and male infertility [98]. Four studies were included in the meta-analysis to evaluate the carbonyl protein (CP) concentration in seminal plasma, containing 197 infertile patients and 190 fertile controls [56, 59, 65, 66]. A fixed-effect model was performed as there was no evidence of heterogeneity among these studies (I^2^ = 13%). And, significantly higher CP concentration was observed in the infertile patients than that in controls (SMD = 2.09, 95% CI: 1.84, 2.34, Z = 16.31, *p* < 0.00001; Figure 16).

### Meta-analysis of glutathione S-transferase activity

**Figure 17:**
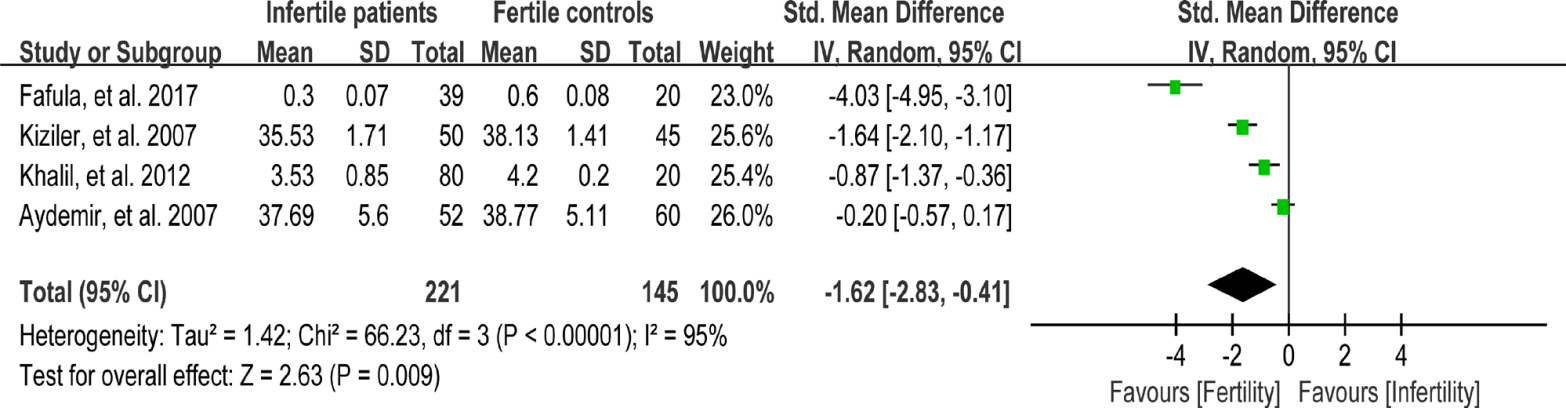
Meta-analysis of studies addressing GST activity in seminal plasma of infertile patients and control subjects Results are shown as standardized mean differences (SMDs).

Glutathione S-transferase (GST) functions to detoxify xenobiotics by catalyzing the nucleophilic which is attacked by GSH on electrophilic carbon, sulfur, or nitrogen atoms of said nonpolar xenobiotic substrates, thereby preventing their interaction with crucial cellular proteins or nucleic acids [99]. Reduced activity of GST may lead to sperm membrane damage, resulting in male infertility [66]. Seminal plasma GST activity was analyzed in meta-analysis with 4 articles containing 221 infertile patients and 145 fertile controls [27, 48, 65, 66]. The heterogeneity was observed (*I*^2^ = 95%), so the integrated effect was calculated by a random-effect model. The results indicated that the GST activity was lower in infertile patients than that in fertile controls (SMD = –1.62, 95% CI: –2.83, –0.41, Z = 2.63, *P* = 0.009 < 0.05; Figure 17).

## DISCUSSION

High ROS levels and oxidative stress have been implicated in the pathophysiology of male infertility and correlated with sperm DNA damage, impaired fertilization and embryo development, low rates of implantation and occurrence of miscarriage [100, 101]. In spite of abundant studies related, it is not well defined how changes in different antioxidants in seminal plasma are correlated with male infertility, and contradictory conclusions could always be found. This may due to the low number of patients and different types of infertility included in these published studies. However, a large-scale systematic study that could address this query is not available, so we reviewed existing literatures and performed a meta-analysis on research studies that reported male infertility associated oxidative stress markers in seminal plasma. With 11 oxidative stress markers obtained from 52 individual studies that included a total of 1428 fertile controls and 2563 infertile patients, our meta-analysis may shed some light into this important issue.

ROS/RNS, as major causes of oxidative stress, plays an important role in many physiological cell processes under a certain concentration, and produce adverse modifications to cell components, such as lipids, proteins, and DNAs. Meta-analysis of these studies has shown that the concentration of seminal NO was significantly increased in infertility patient when compared with fertility controls. Few reports addressed the ROS concentration in seminal plasma, but many studies that had detected the ROS levels in spermatozoa indicated its higher concentration in infertility patients when compared with control ones [10, 49, 102–105]. Accordingly, we could speculate that the ROS concentration is also higher in the seminal plasma of infertility patients, as ROS could easily diffuses across the sperm plasma membrane [106]. Oxidative damages were evaluated in our meta-analysis, which showed that the by-products of lipid and protein peroxidation, MDA and protein carbonyls, were highly accumulated in seminal plasma of infertility patients. On the other hand, 8-OHdG, a major NDA oxidation product, was rarely reported in seminal plasma. Vatannejad, *et al*. has noticed an increased level of 8-OHdG in seminal plasma of infertility patients [107], but more studies are required. Similarly, the level of 8-isoprostane, a stable end product of oxidized lipids derived from arachidonic acid, in seminal plasma was reported to increase by Khosrowbeygi *et al*. [108]. All these findings demonstrate the evidences of oxidative stress be present in seminal plasma of infertility patients.

To counteract the harmful effects of oxidative stress, some strategies like prevention of damage, repair mechanism to alleviate the oxidative damages, physical protection mechanism against damage, and most importantly, the antioxidant defense mechanisms, are present in semen. Semen antioxidant defenses include a network of compartmentalized antioxidant enzymic and nonenzymic molecules that are usually distributed within the spermatozoa and seminal plasma. In our meta-analysis, both enzymic antioxidants and non-enzymic antioxidants were evaluated. The results indicated that the activities of seminal enzymic antioxidants, such as catalase, GPX and GST, were remarkably decreased in infertility patients, and the concentrations of seminal non-enzymic antioxidants, such as GSH, VC and VE, were obviously lower in infertility patients. All of these changes resulted in a significantly lower total antioxidant capacity (TAC) in infertile seminal plasma. SOD catalyzes the dismutation of the superoxide (O_2_^−^) radical into either ordinary molecular oxygen (O_2_) or hydrogen peroxide (H_2_O_2_) [90]. Surprisingly, as an important antioxidant defense in nearly all living cells exposed to oxygen, it shows no statistically difference of activity in seminal plasma of infertility patients and fertility controls according to our meta-analysis. This result suggested that, compared with other antioxidants, the activity of SOD may vary greatly in different kinds of infertility, or more closely related to the severity of the infertility, so the SOD activity may not a suitable assay for infertility diagnosis. Coenzyme Q10 (CoQ10) appears to play an important role in energy metabolism, as well as function as a liposoluble chain-breaking antioxidant for cell membranes and lipoproteins [109]. The level of CoQ10 in seminal plasma was reported to be decreased in infertile patients [55], yet no differences were found between patients and controls in another study [41]. In general, the deficiencies of both enzymic antioxidants and non-enzymic antioxidants could be the exact causes of oxidative stress in the seminal plasma of infertility patients.

The strengths of our current systematic review and meta-analysis include the wide range of markers in oxidative stress evaluated, and the large number of subjects included. However, our study is not free of limitations. First, the heterogeneity among the studies of most analyzed markers (MDA, TAC, SOD, CAT, GPX, GSH, GST, VE) associated with infertility was modest (>50%) and could not be reduced lower than 50% neither by reasonable exclusion nor subgroup analysis (Data not shown). Such heterogeneity may be related to the following factors: (1) search bias may be present when the inclusion of only English and Chinese articles and the exclusion of articles in other languages; (2) selection bias may be of evidence because no randomized controlled trial was performed in these studies and there were differences in the geographic areas and races of the study subjects, and there were also lack of definition for the review population; (3) measurement bias may occur from the differences in the reagent kits, testing equipment, and assay methods. Second, although the publication bias was evaluated in the markers with more than 10 studies, bias may still be present in the fewer than 10 articles reporting other markers. Such biases can be resolved by increasing the number of included articles in future research. Third, SMD analysis can be applied to data where different measurement scales are reported for the same outcome. However, the limitation of SMD analysis is often confined to <10 subjects per group and more conservative than normalized mean difference (NMD) [110]. Finally, the qualities of the pooled evidence were very low as assessed by the GRADE approach (Supplementary Table 1), thus the results must be interpreted with caution.

In conclusion, as summarized in Table 2, in light of the evidence available at present, our systematic review and meta-analysis indicates that several markers of oxidative stress are abnormal in seminal plasma of men with infertility, indicating these might be associated with male infertility, and more studies addressing these markers should be encouraged. Accordingly, this analysis might guide studies of certain interventions upon male infertility.

**Table 2:** Summary of the meta-analysis of oxidative stress markers in seminal plasma of male infertility patients

Marker	Action	Changes in male infertility
MDA	By-product of lipid peroxidation	↑
TAC	Prevents oxidation and detoxifies oxidants	↓
SOD	Converts superoxide anions to hydrogen peroxide and molecular oxygen	↔
Catalase	Detoxifies both intracellular and extracellular H2O2 to water and oxygen	↓
GPX	Reduce lipid hydroperoxides to their corresponding alcohols and to reduce free hydrogen peroxide to water	↓
GST	Detoxify xenobiotics by catalyzing the nucleophilic attack by GSH on electrophilic carbon	↓
GSH	Detoxifies hydrogen peroxide and lipid peroxides, prevents protein from oxidation	↓
NO	Promotes reactive nitrogen species	↑
Carbonyl protein	Products of protein oxidation	↑
VC	Neutralize hydroxyl, superoxide, and hydrogen peroxide radicals	↓
VE	scavenge superoxide, H_2_O_2_, and hydroxyl radicals	↓

↓, decreased; ↑, increased; ↔, unchanged.

## SUPPLEMENTARY MATERIALS FIGURES AND TABLE


