# Supplementary material for: Is male infertility associated with increased oxidative stress in seminal plasma? A-meta analysis
Source: Oncotarget. 2018 May 11;9(36):24494–513. doi: 10.18632/oncotarget.25075 (PMC5966266; doi:10.18632/oncotarget.25075)
Supplement: Supplementary file 1 [file oncotarget-09-24494-s001.pdf]

Is male infertility associated with increased oxidative stress in seminal plasma? A-meta analysis

SUPPLEMENTARY MATERIALS

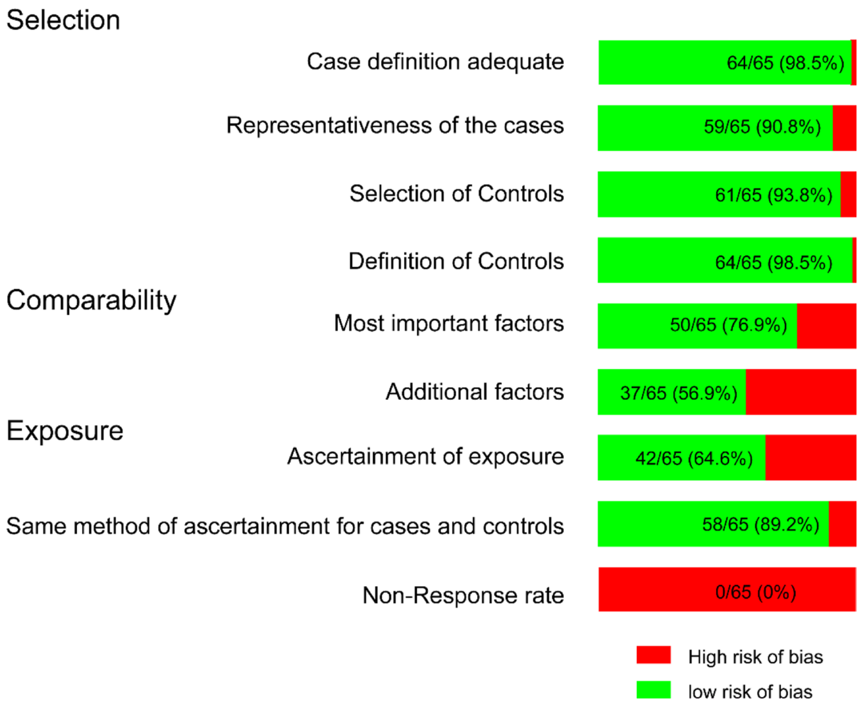

Supplementary Figure 1: Risk of bias by domain (in bold) and question in nine case-control studies using the Newcastle–Ottawa Scale. Numbers on the green bar represent the number of studies with low risk of bias over the number of studies assessed.

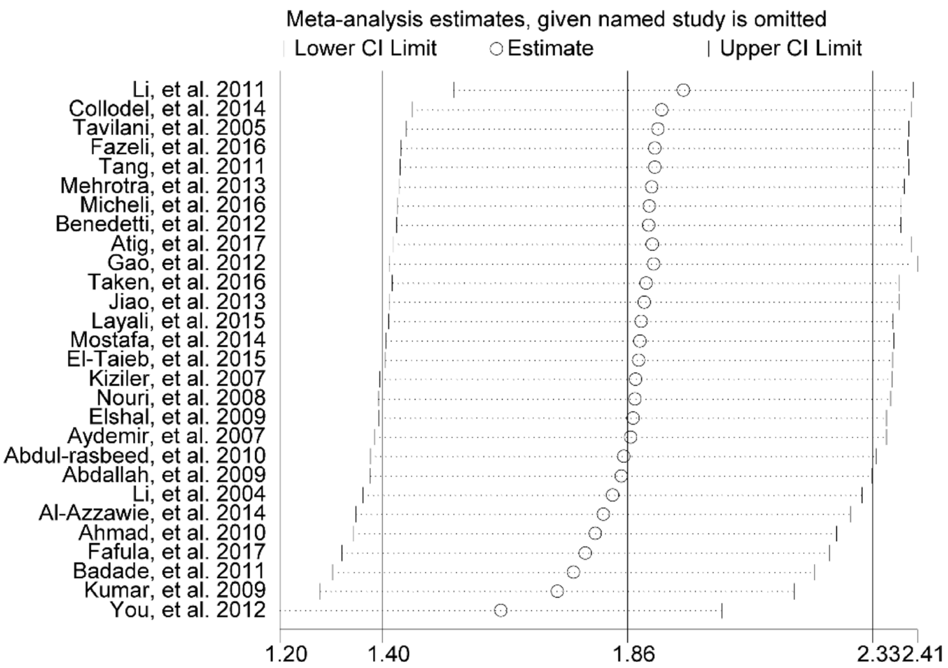

Supplementary Figure 2: Sensitivity analysis was performed to evaluate the robustness of conclusion addressing the risk factor of MDA.

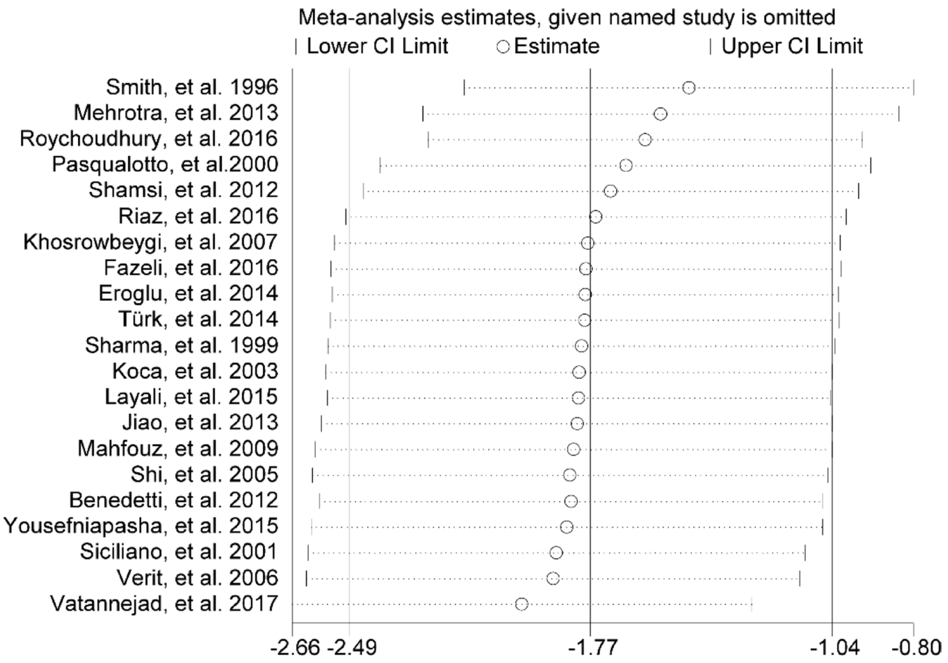

Supplementary Figure 3: sensitivity analysis was performed to evaluate the robustness of conclusion addressing the risk factor of TAC.

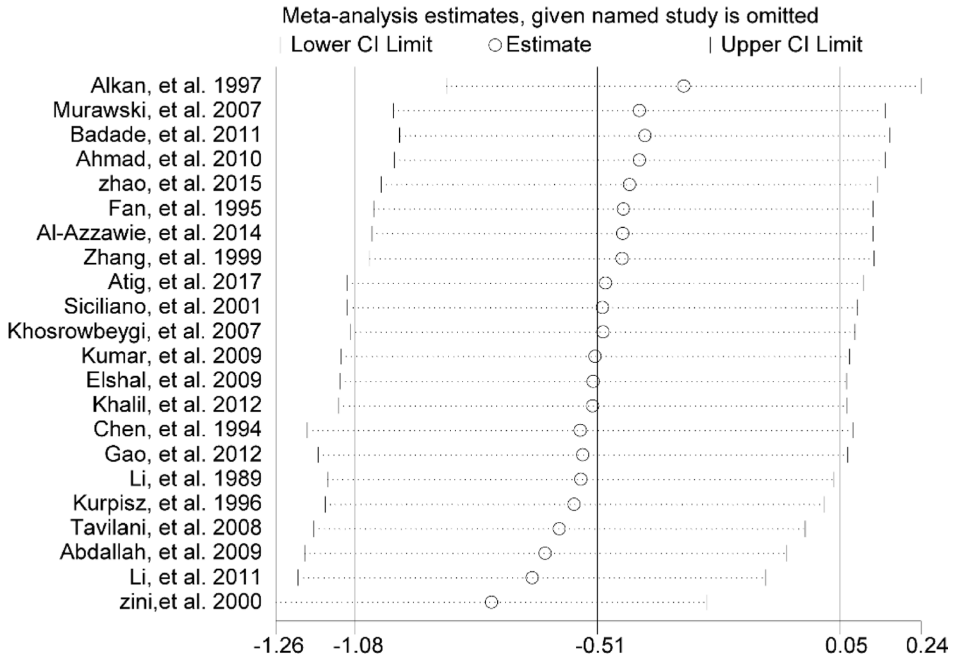

Supplementary Figure 4: Sensitivity analysis was performed to evaluate the robustness of conclusion addressing the risk factor of SOD.

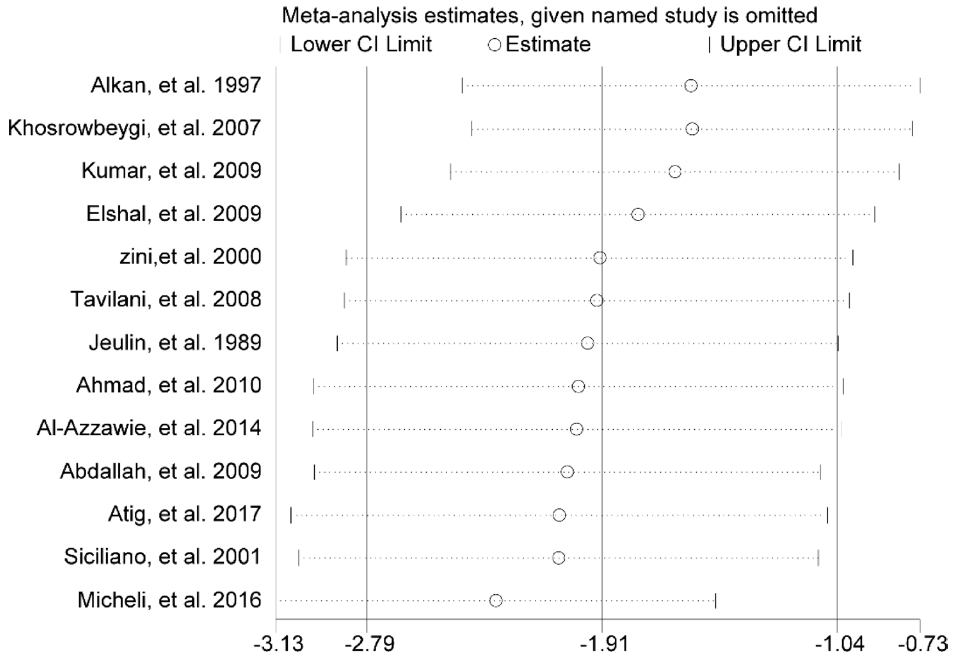

Supplementary Figure 5: Sensitivity analysis was performed to evaluate the robustness of conclusion addressing the risk factor of catalase.

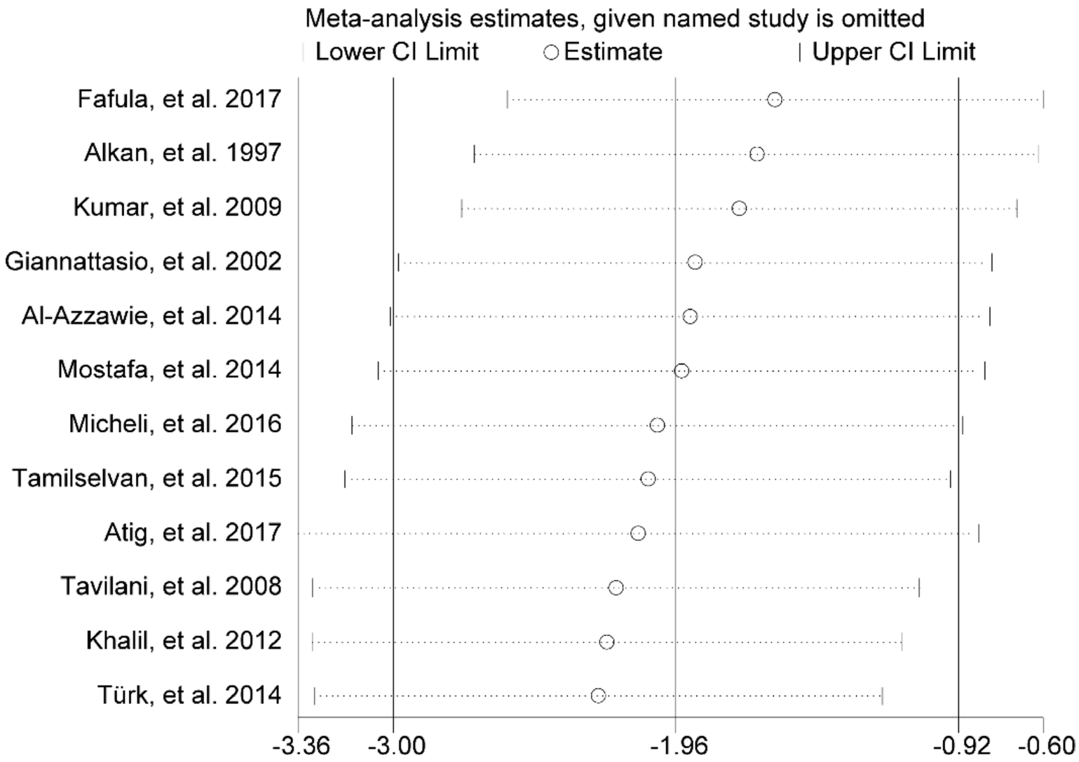

Supplementary Figure 6: Sensitivity analysis was performed to evaluate the robustness of conclusion addressing the risk factor of GPX.

Supplementary Table 1: Summary of findings for the main comparison

Outcomes	Illustrative comparative risks* (95% CI)		Relative effect (95% CI)	No of Participants (studies)	Quality of the evidence (GRADE)	Comments
	Assumed risk	Corresponding risk				
MDA concentration	See comment	See comment		2283 (28 studies1)	⊕⊝⊝⊝ very low[2,3]	1412 cases and 871 controls in case-control studies
Total antioxidant capacity	See comment	See comment		1808 (21 studies1)	⊕⊝⊝⊝ very low[2]	1255 cases and 553 controls in case-control studies
Superoxide dismutase activity	See comment	See comment		2568 (22 studies1)	⊕⊝⊝⊝ very low[2]	1808 cases and 760 controls in case-control studies
Catalase activity	See comment	See comment		1003 (13 studies1)	⊕⊝⊝⊝ very low[2]	740 cases and 263 controls in case-control studies
Glutathione peroxidase activity	See comment	See comment		738 (11 studies1)	⊕⊝⊝⊝ very low[2,3]	458 cases and 280 controls in case-control studies
GSH concentration	See comment	See comment		633 (8 studies1)	⊕⊝⊝⊝ very low[2]	388 cases and 245 controls in case-control studies
Nitric oxide concentration	See comment	See comment		548 (6 studies1)	⊕⊝⊝⊝ very low[2]	326 cases and 222 controls in case-control studies
Vitamin E concentration	See comment	See comment		432 (5 studies1)	⊕⊝⊝⊝ very low[2]	257 cases and 175 controls in case-control studies
Vitamin C concentration	See comment	See comment		389 (4 studies1)	⊕⊝⊝⊝ very low[4]	226 cases and 163 controls in case-control studies
Carbonyl protein concentration	See comment	See comment		387 (4 studies1)	⊕⊝⊝⊝ very low[4]	197 cases and 190 controls in case-control studies

GRADE Working Group grades of evidence

High quality: Further research is very unlikely to change our confidence in the estimate of effect.

Moderate quality: Further research is likely to have an important impact on our confidence in the estimate of effect and may change the estimate.

Low quality: Further research is very likely to have an important impact on our confidence in the estimate of effect and is likely to change the estimate.

Very low quality: We are very uncertain about the estimate.

[1]case-control.

[2]With notable statistical heterogeneity.

[3]Egger's regression analysis displayed an evidence of publication bias.

[4]Total population size is less than 400.
